# Beat-Level Interpretation of Intra-Patient Paradigm Based on Object Detection

**DOI:** 10.3389/fcvm.2022.857019

**Published:** 2022-03-18

**Authors:** Man Kang, Xue-Feng Wang, Jing Xiao, He Tian, Tian-Ling Ren

**Affiliations:** ^1^The School of Integrated Circuits, Tsinghua University, Beijing, China; ^2^The Beijing National Research Center for Information Science and Technology, Tsinghua University, Beijing, China; ^3^Ping An AI Research Center, Ping an Technology (Shenzhen) Co. Ltd., Shenzhen, China

**Keywords:** object detection, ECG, beat-level classification, deep learning, automatic annotation

## Abstract

Electrocardiogram (ECG), as a product that can most directly reflect the electrical activity of the heart, has become the most common clinical technique used for the analysis of cardiac abnormalities. However, it is a heavy and tedious burden for doctors to analyze a large amount of ECG data from the long-term monitoring system. The realization of automatic ECG analysis is of great significance. This work proposes a beat-level interpretation method based on the automatic annotation algorithm and object detector, which abandons the previous mode of separate R peak detection and heartbeat classification. The ground truth of the QRS complex is automatically annotated and also regarded as the object the model can learn like category information. The object detector unifies the localization and classification tasks, achieving an end-to-end optimization as well as decoupling the high dependence on the R peak. Compared with most advanced methods, this work shows superior performance. For the interpretation of 12 heartbeat types in the MIT-BIH dataset, the average accuracy is 99.60%, the average sensitivity is 97.56%, and the average specificity is 99.78%. This method can be used as a clinical auxiliary tool to help doctors diagnose arrhythmia after receiving large-scale database training.

## Introduction

The WHO report regards cardiovascular disease as one of the leading causes of death worldwide, and it will continue to exist in the near future. The burden of cardiovascular disease is so heavy that research on heart health has to be taken seriously. Electrocardiogram (ECG), as a product that can most directly reflect the electrical activity of the heart, has become the most common clinical technique used for the analysis of cardiac abnormalities. However, it is a heavy and tedious burden for doctors to analyze a large amount of ECG data from the long-term monitoring system. It is necessary to realize automatic ECG analysis.

**Figure 1:**
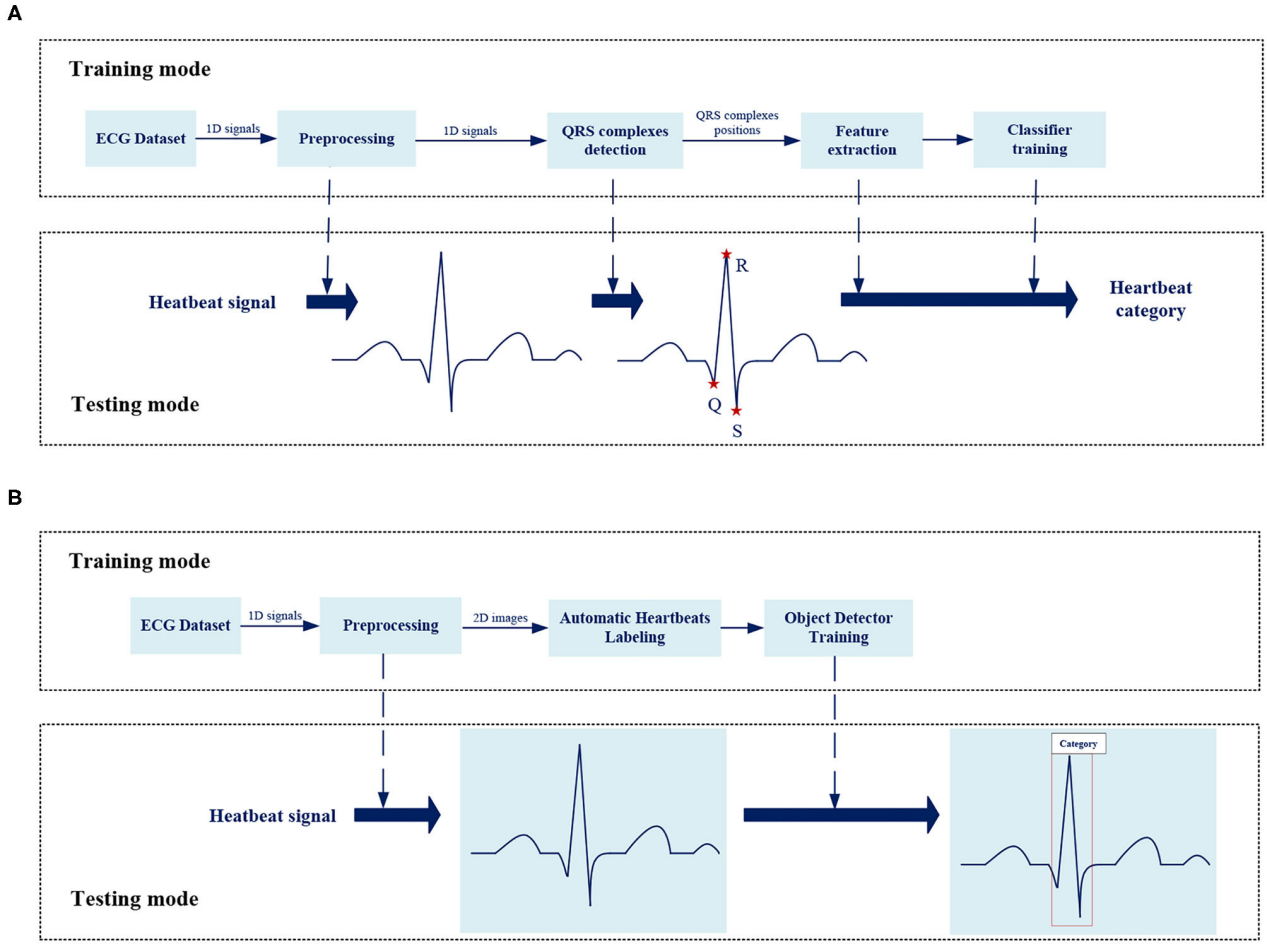
The overview of the framework for analysis of ECG abnormality. **(A)** The framework of traditional method for ECG analysis. **(B)** The framework of proposed method for ECG analysis.

In the past few decades, the open source ECG databases have promoted the development of automatic ECG analysis. Most methods are based on one-dimensional ECG signals, as shown in Figure 1A, which mainly involve four steps: signal preprocessing, R-peak detection, feature extraction, and classifier construction.

### R-Peak Detection

Over the years, a large number of studies have made efforts in QRS complex detection. Pan and Tompkins (1) realized the automatic detection of the R-peak through the first derivative, non-linear transformation and amplitude/noise threshold. Using time-domain features, Yeh and Wang (2) proposed a differential operation method (DOM) algorithm. Li et al. (3) introduced wavelet transform to the automatic detection of R-peak for the first time. Martinez et al. (4) remove the singularity analysis, and consider any possible QRS complex wave shape, search for positive and negative zero crossing points. Manikandan and Soman (5) introduced a new method using Shannon energy estimation and Hilbert transform. These methods are time-consuming, which require complex mathematical calculations, as well as the accuracy is not always ideal.

### Heartbeat Classification

According to different feature extraction methods, heartbeat classification can be divided into artificial methods and automatic methods. Common artificial features include morphological features (6–8) such as RR interval, and ECG transform coefficients (9–13) such as wavelet transform. These features are sent to a traditional classifier for interpretation. For example, Hu (14) extracted features based on multiple discriminant and principal component analysis, and used support vector machine (SVM) for classification. Song et al. used linear discriminant analysis combined with SVM to analyze six types of arrhythmias (15). Melgani and Bazi proposed an SVM classifier based on particle swarm optimization (16). Martis et al. used a four-layer feedforward neural network and a least squares support vector machine (LS-SVM) to divide heart beats into five categories (17). Ganeshkumar and Kumaraswamy introduced a random forest tree (RFT) as a classifier (18). Park et al. proposed a K-nearest neighbor (K-NN) classifier (19). Jun et al. proposed a parallel K-NN classifier for high-speed arrhythmia detection (20). The accuracy of the above methods highly depends on the effectiveness of feature extraction, which requires strong scientific theories and doctors' personal experience as support, as well as the computational complexity is high. With the development and application of deep learning technology, it has also become a research hotspot in the field of ECG classification. In some previous studies, the simple 1D-CNN was used to classify a time series of ECG signals (21–23) or one-dimensional heartbeats (24). Acharya et al. proposed a nine-layer deep CNN (25), which can identify five different heartbeats. Chauhan and Vig used the deep long short-term memory (LSTMs) network to classify abnormal and normal signals (26). Eltrass et al. (27) proposed the CQ-NSGT algorithm, a new method for converting one-dimensional signals into time-frequency maps, and used AlexNet for time-frequency maps classification. Warrick and Homosi proposed a new method to automatically classify arrhythmias in ECG, using a combination of CNN and LSTM (28). Shu et al. also proposed a system with combination of CNN and LSTM (29) to identify five heart beats. Wong et al. (30) proposed a FPGA implementation of ECG classifier based on bCNN, the core of which is to reduce the computational complexity of CNN. Yao et al. (31) proposed an integrated CNN and GRU classifier to classify a time series of ECG signals. Sarin et al. (32) compared the classification accuracy of MLP, CNN, and LSTM on a subset of the MIT-BIH dataset.

Most previous works just classify a signal over a long period of time to get simple information about the existence of abnormality. There are many types of heartbeats in a section of ECG signal such as bigeminy and trigeminy, so this work focuses on the interpretation of beat-level to get detailed information about each beat for further analysis. Beat-level classification in the past required the R-peak detection before classification especially the inference stage also cost the same as the training mode. More importantly, the classification accuracy was highly dependent on R-peak detection quality and the independence of R-peak detection made end-to-end optimization impossible. In this work, the position of QRS complex is also taken as the object to be learned by the model-like category information, which unifies the positioning and classification tasks based on the object detector, achieving an end-to-end overall optimization without independent time-consuming R-peak detection process. Besides, we expect the model to classify by learning features from the morphology of heartbeats, much like doctor's eyes, as shown in Figure 1B, this work analyzes the original two-dimensional images of heartbeats, not performing a series of complex mathematical calculations for one-dimensional signals or time-frequency diagram.

## Proposed Method

Figure 1B shows the overall framework of the ECG analysis method in this work. There are two key designs: *Automatic heartbeats annotation* and *Object detector*.

### Signal Preprocessing

The ECG signal has some interference such as baseline drift and high-frequency noise. In order to obtain a better detection effect in the follow-up, it is necessary to preprocess the signal in the early stage.

#### Power Frequency Interference

**Figure 2:**
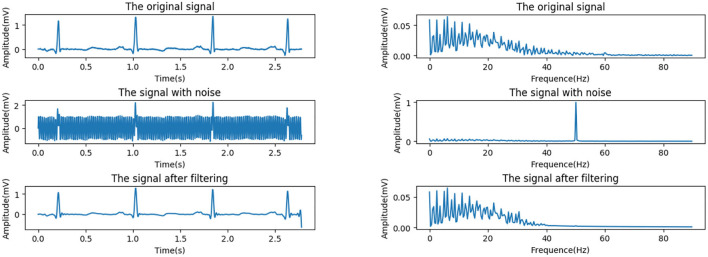
Power frequency interference reduction. **(Left)** Time domain. **(Right)** Frequency domain.

According to the standards published by the American College of Cardiology (ACC), for the normal ECG signal, the amplitude range is between 0.05 and 5 mV, the frequency range is within the range of 0.05–100 Hz, and the spectral energy is generally concentrated between 0.25 and 35 Hz. This work uses a Butterworth filter with a cutoff frequency of 45 Hz and order of 10 to remove power frequency interference. In order to evaluate the effect of noise reduction, we add a 50 Hz noise signal to simulate power frequency interference. Figure 2 shows the comparison of the effect before and after the noise signal processing.

#### Baseline Drift

Baseline drift is generally caused by human breathing and electrode movement, which belongs to low-frequency interference, usually below 0.5 Hz. The ST-segment frequency band is in the range of 0.7–2.0 Hz, which partially overlaps with the baseline drift frequency band, 0.05–1.5 Hz. It is necessary to avoid obvious deformation of low frequency parts such as ST segment, or which will lead to detection and analysis distortion. Therefore, the median filter, which has a good effect on suppressing larger drift as well as protecting smaller P-wave and T-wave, is adopted.

The effect of drift removal mainly depends on the filter window width. If the width is too small, the fitted baseline has more high-frequency components, while too large width causes too much computation and affects speed of the algorithm. Considering both speed and effect, as well as for convenience of value, the width is set to half of the sampling rate.

Besides, in order to avoid edge effects, the signal is expanded before filtering. Specifically, the endpoint value instead of 0 is added to both ends of the signal to avoid larger errors.

**Figure 3:**
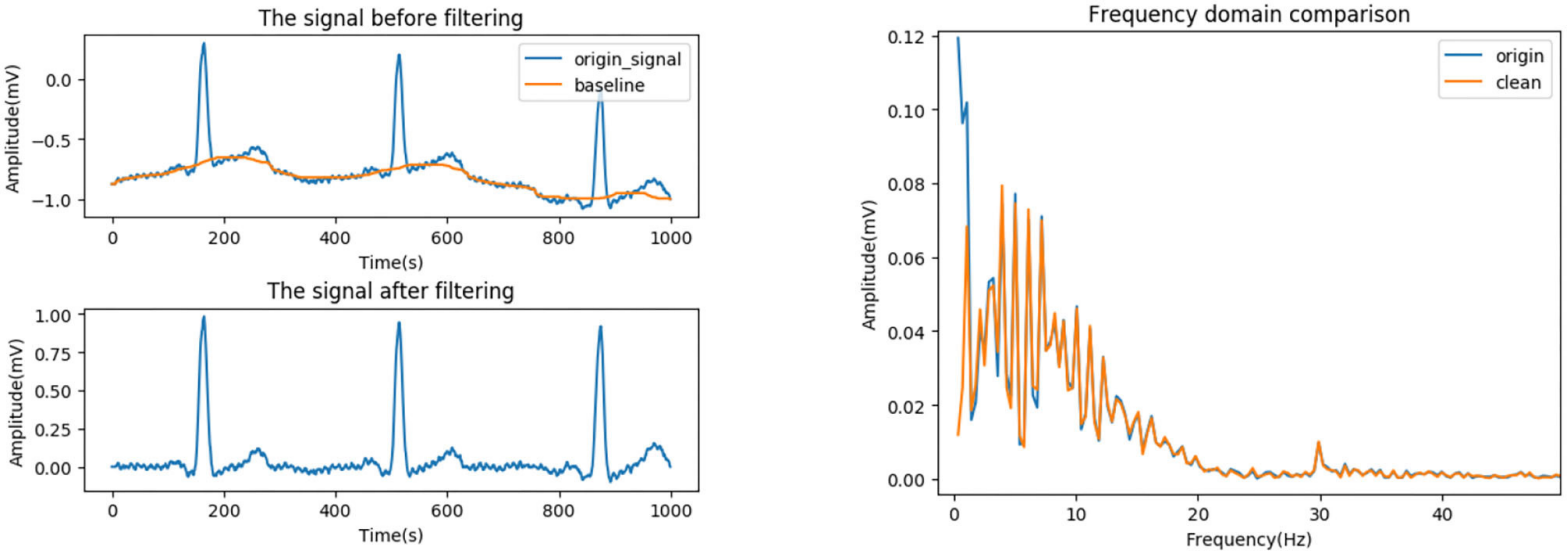
Baseline drift removing. **(Left)** Time domain. **(Right)** Frequency domain.

Figure 3 shows the effects before and after the processing of a signal with severe baseline drift in the MIT-BIH database (see section MIT-BIH Database). It can be seen that the drift is well-suppressed, and only the very low-frequency part is attenuated while the high-frequency part is barely affected.

### Automatic Heartbeats Annotation

After the signal preprocessing described in section Signal Preprocessing, the heartbeat images are saved in*.jpg* format. In order to take the position information as the object that can be learned by the subsequent model, it is necessary to annotate the location of the heartbeats.

The transient degree of signal is often described by singularity. Wavelet transform is an effective method to analyze signal singularity, which has promising features for characterizing the local regularity of signals by decomposing the signal into elementary building blocks that are well-localized both in time and frequency (33). Each pair of positive and negative modulus maximum of the wavelet coefficient corresponds to a signal singularity, and the zero-crossing point between the pair is the singularity position. The relationship between wavelet decomposition and singularity varies in different scales. The small scale reflects the high-frequency component of the signal, while the large scale corresponds to the opposite.

Different bands could be located by wavelet decomposition at different scales due to an ECG signal varies in frequency. Q-wave and S-wave are typical high-frequency waves with low amplitude, whose energy is concentrated in small scale.

**Figure 4:**
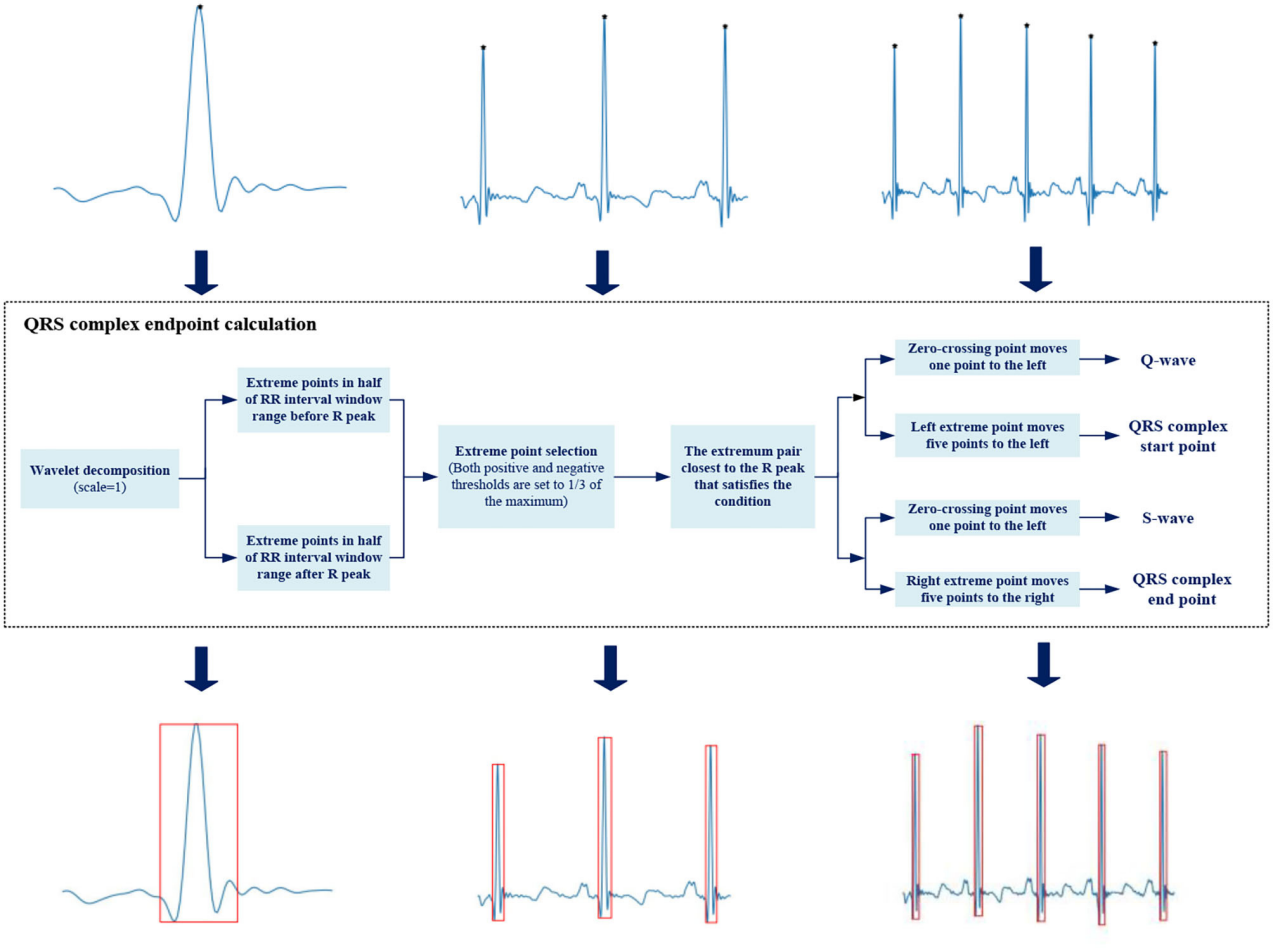
Automatic heartbeats annotation.

We select the wavelet coefficients at *j* = 1 scale (34), and the officially marked R peaks are used as the reference to locate Q-wave and S-wave. The R peak corresponds to the zero-crossing point of the modulus maximum–minimum pair, Q-wave and S-wave are located at the modulus minimum and maximum within a certain range before and after the R peak, respectively. We need to give certain delay compensation during the actual operation because each waveform is not completely symmetric. Figure 4 shows the detailed process.

We use a rectangular box to annotate the position of QRS complex, with Q-wave position as the left boundary, S-wave position as the right boundary, R-peak position as the upper boundary, and the smaller ordinate in Q-wave and S-wave as the lower boundary. The upper left corner *(xmin, ymin)* and lower right corner *(xmax, ymax)* are saved as location information in .*xml* file format.

### Object Detector

**Figure 5:**
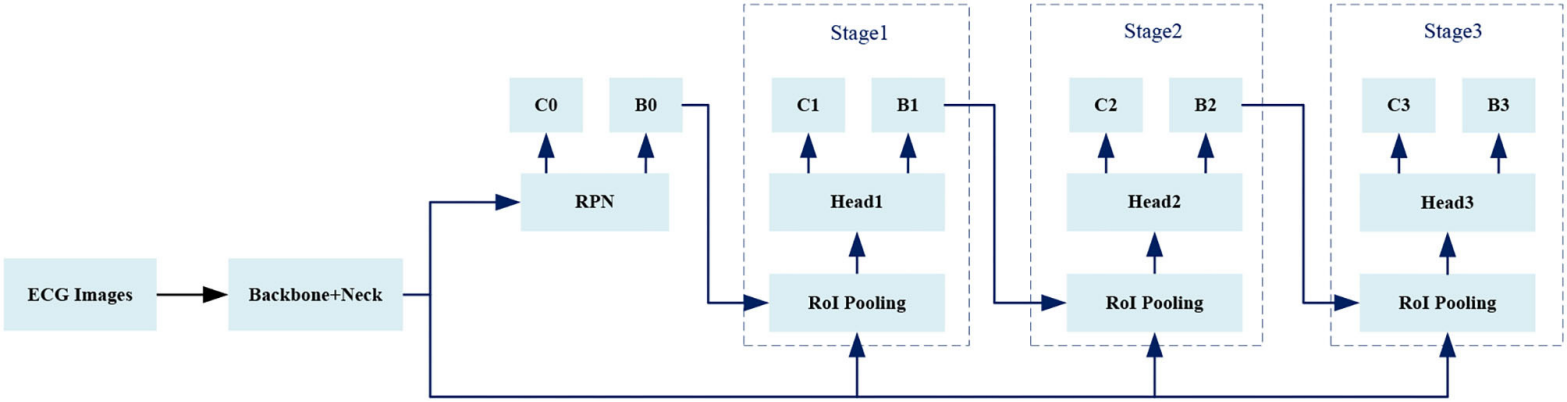
The framework of object detector.

We use *Cascade RCNN* (35). as a basic model, and Figure 5 shows the overall framework of the model.

#### Region Proposal Network

**Figure 6:**
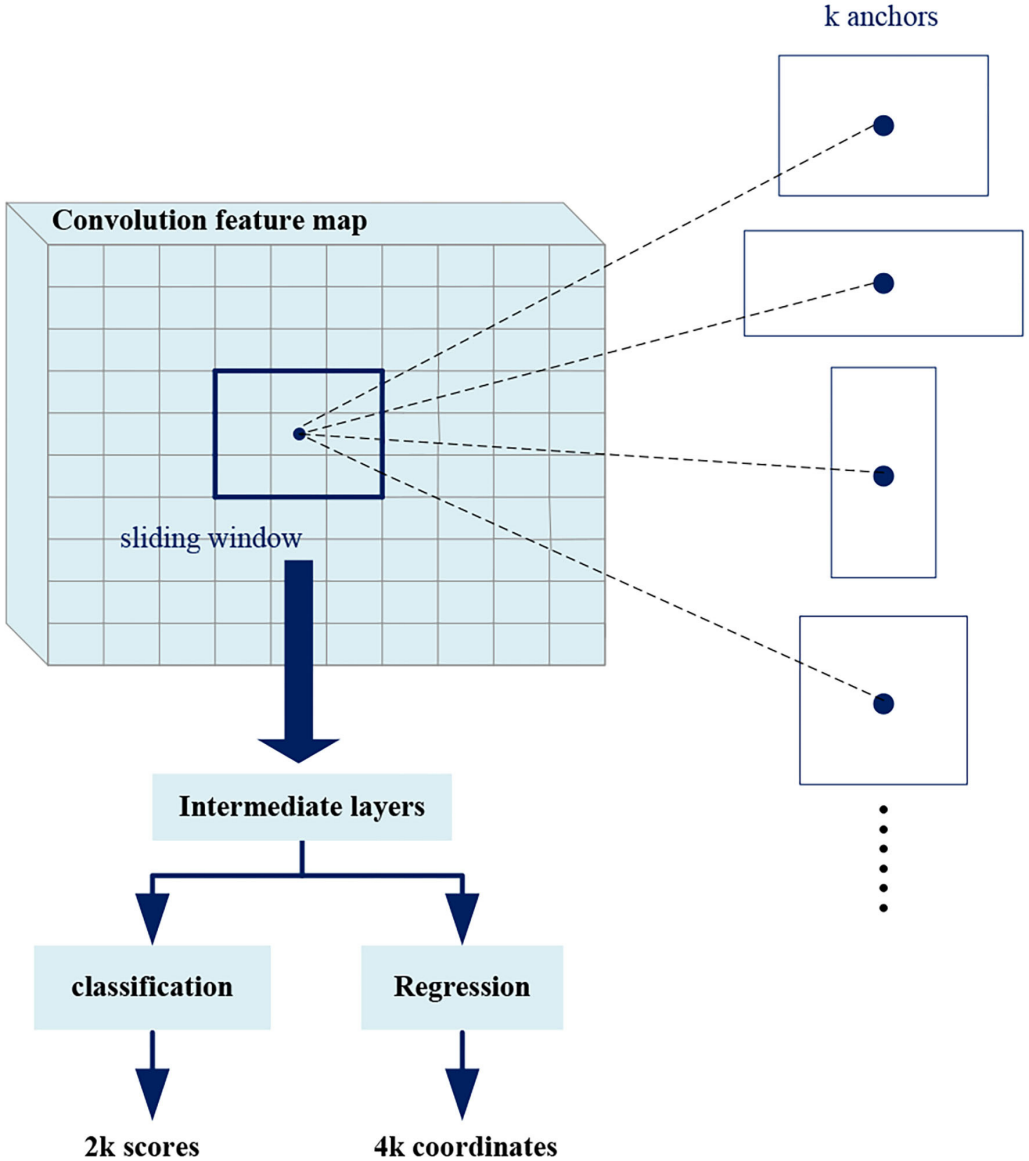
Region proposal network (RPN).

Region proposal network takes an image as input and outputs in a set of rectangular boxes called proposals. Each proposal has a score, which measures the confidence belongs to foreground. As shown in Figure 6, in order to generate a region proposal, we slide a small window of n^*^n (*n* = 3 in this work) on the convolutional feature map. Each sliding window is mapped to a lower-dimensional feature that is fed into two fully connected layer branches: a regression branch (*reg*) and a classification branch (*cls*). Since it operates in a sliding window mode, the fully connected layers are shared in all spatial locations.

At each sliding window position, we predict multiple region proposals at the same time, where the maximum number of proposals at each position is denoted as *k* (*k* = *3*). Therefore, the *reg* branch has *4k* outputs to encode the coordinates of the *k* rectangular boxes, and the *cls* branch outputs *2k* scores to estimate the foreground or background probability of each proposal. The *k* proposals are parameterized with respect to *k* reference boxes called anchors. The anchors center on the sliding window and are associated with scale and aspect ratio. In order to reduce redundancy, Non-maximum suppression (NMS) is used for proposals according to their scores. Then the proposals with top scores are selected for follow-up detection. The *NMS* is explained in detail later.

#### Region of Interest Pooling

Region of interest *pooling* uses maximum pooling to transform the features in *RoI* into a small feature map of fixed size H^*^W (7^*^7), where *H* and *W* are hyperparameters independent of any specific *RoI*. The inputs of *RoI pooling* are coordinate information of the proposals from *RPN* or that of the predicted boxes from previous stage, and the convolution feature map of a certain layer or several layers.

**Figure 7:**
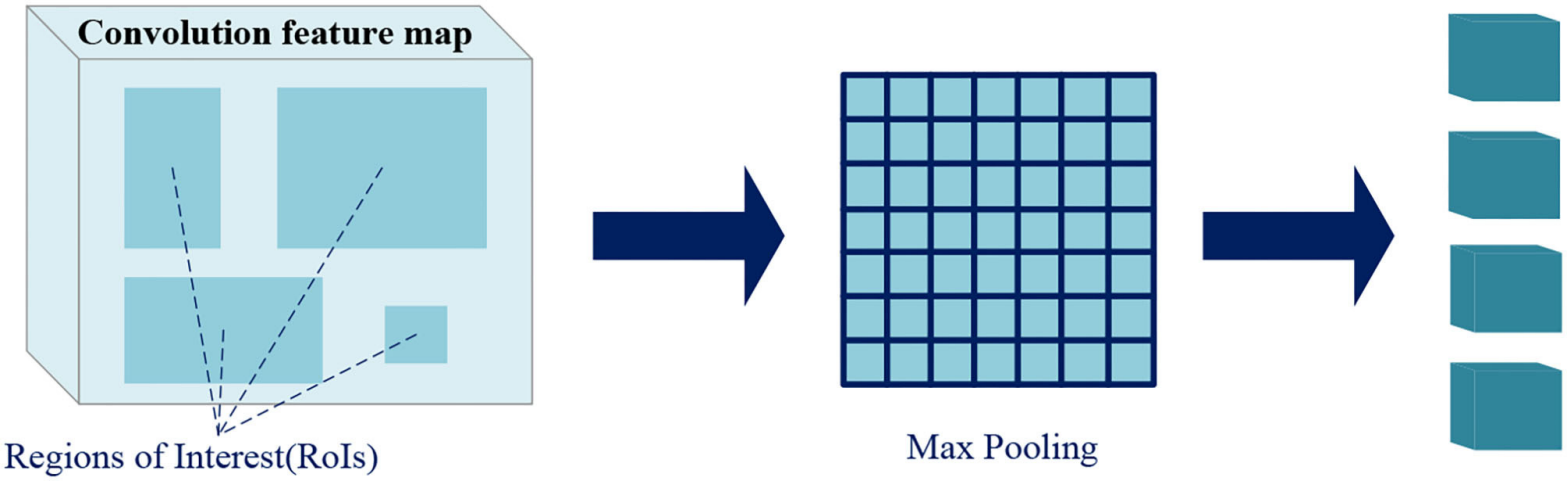
Region of interest (RoI) pooling.

As shown in Figure 7, the *RoI pooling* layer maps the coordinates to the corresponding position in the feature map. Each *RoI* is a rectangular area in the convolutional feature map, which is defined by a quadruple *(x, y, h, w)*, respectively, corresponding to its center point coordinates *(x, y)*, height *h*, and width *w*. *RoI pooling* divides the *RoI* of h^*^w into H^*^W grids consisted of sub-windows of approximate size h/H^*^w/W, and performs maximum pooling operation on each sub-window. Pooling is applied to each feature map channel independently, just like standard maximum pooling, thus we get fixed size feature maps from *RoIs* of varying sizes.

#### Head

The *Head* layer is responsible for further processing the fixed size feature maps from *RoI pooling* to output the final detection results. Each feature map will go through a series of fully connected layers, and finally branch into two output layers, classification and regression. For each *RoI*, the classification layer outputs the softmax probabilities of *C* foreground classes and one background class, while the regression layer outputs four real numbers encoding predicted boxes position.

#### Loss Function

In order to calculate the loss of each predicted box, we need to classify them as foreground or background according to their Intersection over Union (IoU) with ground truths. *IoU* will be described in detail later.

Given an *IoU* threshold *IoU_thres*, the predicted boxes which have maximum *IoU* with ground truths and the predicted boxes whose *IoU* with any ground truth greater than *IoU_thres* are regarded as foregrounds, while the predicted boxes whose *IoU* with all ground truths less than *IoU_thres* are backgrounds. For Stage1, Stage2, and Stage3 in Figure 5, the value of *IoU_thres* is increasing.

A bounding box is encoded as (45),


(1) $$\begin{array}{r} \begin{array}{c} t_{x}=\left(x-x_{a}\right)/w_{a},\;t_{y}=\left(y-y_{a}\right)/h_{a},\\ t_{w}=\log\left(w/w_{a}\right),\;t_{h}=\log\left(h/h_{a}\right),\\ t_{x}^{*}=\left(x^{*}-x_{a}\right)/w_{a},\;t_{y}^{*}=\left(y^{*}-y_{a}\right)/h_{a},\\ t_{w}^{*}=\log\left(w^{*}/w_{a}\right),\;t_{h}^{*}=\log\left(h^{*}/h_{a}\right) \end{array} \end{array}$$

where *x, y, w, h* are the center point's coordinates, width, and height of a rectangular box, *x, x*_*a*_, x^*^, respectively, correspond to the predicted box, anchor and ground truth, *y, w, h* are similar.

The location information we annotate are upper left corner *(xmin, ymin)* and lower right corner *(xmax, ymax)* of a rectangular box. So before calculating the regression loss, we convert the coordinates to a quadruple *(x, y, h w)*, where

*x*=*(xmin* + *xmax)/2, y*=*(ymin* + *ymax)/2, w*=*xmax-xmin, h*=*ymax-ymin*,

Then the four values are encoded by Equation (1), which are used as ground truth labels.

The regression loss of a predicted box is defined as


(2) $$\begin{array}{r} L_{reg}\left(t,t^{*}\right)=\underset{i\in \left\{x,y,w,h\right\}}{\sum }smooth_{L1}\left(t_{i}-t_{i}^{*}\right) \end{array}$$

where


(3) $$\begin{array}{r} \text{s}mooth_{L1}(x)=\{\begin{array}{c} 0.5x^{2},if\left|x\right|<1\\ \left|x\right|-0.5,otherwise \end{array} \end{array}$$

The classification loss is calculated by cross entropy, which is defined as


(4) $$\begin{array}{r} L_{cls}\left(p,c\right)=-\log p_{c} \end{array}$$

where *p* is the softmax probability vector from the classification branch, *c* is the true category label, taking 0, 1, 2...C. C is the number of foreground classes, and 0 corresponds to the background class.

The loss function of each stage is defined as (35).


(5) $$\begin{array}{r} L_{\text{s}}=\underset{n}{\sum }L_{cls}\left(p_{s,n},p_{s,n}^{*}\right)+\lambda \underset{n}{\sum }p_{s,n}^{*}L_{reg}\left(t_{s,n},t_{s,n}^{*}\right) \end{array}$$

where balances the weight of classification loss and regression loss, only the boxes belong to foreground class $p_{s,n}^{*}=1$ need to be calculated for regression losses.

The total loss function is the sum of the three stages


(6) $$\begin{array}{r} L=L_{1}+L_{2}+L_{3} \end{array}$$

#### Non-Maximum Suppression

Non-maximum suppression, which is usually used in the *RPN* and inference stage, aims to extract predicted boxes with high confidence and suppresses the predicted boxes with low confidence, thus could remove redundant boxes detecting the same object.

**Figure 8:**
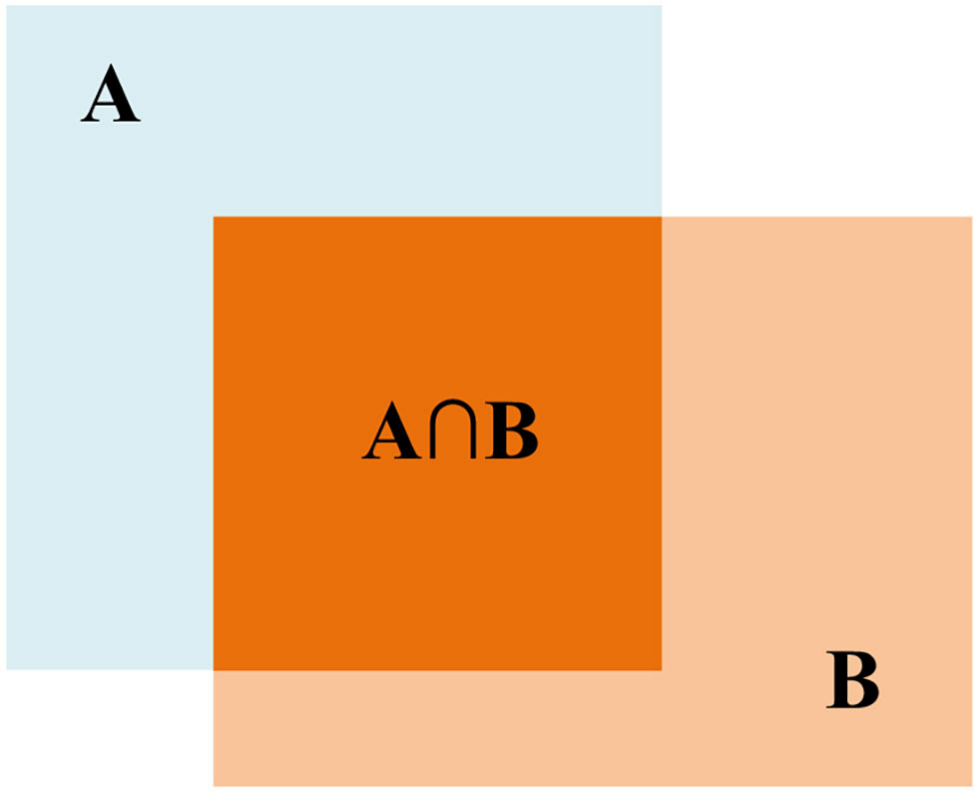
Intersection over union (IoU).

Before describing *NMS*, it is necessary to explain the IoU, which measures the overlap between two boxes. As shown in Figure 8, the *IoU* of box A and box B is


(7) $$\begin{array}{r} IoU=\frac{A⋂B}{A⋃B} \end{array}$$

Obviously, the higher the *IoU* value, the higher the degree of overlap between two boxes.

For an image, the final classification branch and regression branch will output confidence scores and coordinate information of a series of boxes. The specific process of the *NMS* is as follows. *B* represents the box set. First, selecting the predicted box *M* with the maximum confidence score and adding it to the set *D* representing final results. Second, removing the box *M* and other category boxes whose *IoU* with *M* exceed preset threshold from *B*. Next, repeating the two steps until *B* is empty. In this work, only the boxes with the highest confidence score can be regarded as the final predicted results in *NMS* because there is no overlap between different heartbeat objects.

## Experiments and Results

### MIT-BIH Database

MIT-BIH arrhythmia database, one of the four internationally recognized standard ECG databases, is used for experimental evaluation in this work. The database contains a total of 48 records from 47 different patients, and the ECG signals are sampled at 360 Hz with 11-bit resolution.

Each record corresponds to about 30 min of ECG data, including signals from two leads. For all records, the first lead is the modified lead II (MLII) and the second lead is V1, V2, V4, or V5. Twenty-three records are randomly selected from 4,000 continuous 24-h dynamic ECG signals of patients at Beth Israel Hospital (BIH), while the remaining data are selected from a few rare ECG data samples and have important clinical significance. The notes are authoritatively certified by multiple cardiologists.

The Association for the Advancement of Medical Instrumentation (AAMI) (46) divides the heartbeats in the MIT-BIH database into five categories. Table 1 shows the specific categories and numbers.

**Table 1:** Categories and numbers of beats in the MIT-BIH database.

**AAMI classes**	**MIT-BIH Annotations**	**Description**	**Numbers**
Normal(N)	N	Normal beat	75,052
	L	Left bundle branch block beat	8,075
	R	Right bundle branch block beat	7,259
Supraventricular ectopic beat (SVEB)	E	Atrial escape beat	16
	J	Nodal (junctional) escape beat	229
	A	Atrial premature beat	2,546
	A	Aberrated atrial premature beat	150
	J	Nodal (junctional) premature beat	83
	S	Supraventricular premature or ectopic beat (atrial or nodal)	2
Ventricular ectopic beat (VEB)	V	Premature ventricular contraction	7,130
	E	Ventricular escape beat	106
Fusion (F)	F	A fusion of ventricular and normal beat	803
Unknown beat (Q)	/	Paced beat	7,028
	F	A fusion of paced and normal beat	982
	Q	Unclassifiable beat	33

Given *MLII* is the only lead representing all records in this database, as well as the most commonly used lead for experts to analyze, this work only extracts the ECG signals of *MLII* lead for experiments. According to AAMI's recommendation, the rhythm heartbeat records, i.e., 102, 104, 107, and 217, are not used, and the Q category which does not actually exist is also ignored.

In order to balance the number of different heartbeat categories, we only sample a part of *Normal* heartbeats as samples, and perform data augmentation by translating and resizing for various heartbeat images. A total of 60,000 heartbeat images are used in the final experiment, which are divided into training sets and testing sets in a ratio of 8:2. Table 2 shows the numbers for each category in detail.

**Table 2:** Categories and numbers of beats.

**Classes**	**Training set**	**Testing set**
N	4,013	987
L	3,992	1,008
R	3,975	1,025
e	3,994	1,006
j	3,971	1,029
A	4,053	947
a	4,031	969
J	3,985	1,015
S	3,957	1,043
V	4,002	998
E	4,014	986
F	4,013	987
Total	48,000	12,000

### Evaluation Metrics

Consistent with previous research, classification accuracy(*Acc*), sensitivity(*Sen*), and specificity(*Spe*) are used as evaluation metrics in this work.


(8) $$\begin{array}{r} Acc=\frac{TP+TN}{TP+FN+FP+TN} \end{array}$$

(9) $$\begin{array}{r} Sen=\frac{TP}{TP+FN} \end{array}$$

(10) $$\begin{array}{r} Spe=\frac{TN}{TN+FP} \end{array}$$

The above metrics are applicable to each heartbeat category, where *TP* is the number of heartbeats correctly classified as positive samples, *TN* is the number of heartbeats correctly classified as negative samples, *FP* is the number of heartbeats incorrectly classified as positive samples, and *FN* is the number of heartbeats incorrectly classified as negative samples.

### Implementation Details

We use the *Cascade RCNN* equipped with Feature Pyramid Network (FPN) (47) as our default framework, the *ImageNet* pre-trained *ResNet-50* (48) is adopted as the backbone, and the *RoI Align* (49) is used to replace the *RoI pooling*. Our implementation and hyperparameters are based on *MMDetection* (50). Anchors with 1 scale and 3 aspect ratios are used. *NMS* with a threshold of 0.7 is used to generate 2,000 and 1,000 proposals for training and inference. In each training step, 512 proposals are sampled from 2,000 proposals for training, and the ratio of foreground to background proposals is 1:3.

The model is trained for 24 epochs on 4 GPUs with 4 images per GPU. With *SGD* optimizer, the learning rate is initialized to 0.02 and divided by 10 at the 16th and 22nd epoch. The weight decay and momentum are set to 0.0001 and 0.9, respectively.

The optimal model with the highest *mAP@0.5* is obtained by using 5-fold cross-validation during training. Table 3 shows its prediction accuracy of each category on the testing set.

**Table 3:** The results of each category on testing set.

**Classes**	**Acc (%)**	**Sen (%)**	**Spe (%)**
N	99.19	93.52	99.70
L	99.94	99.60	99.97
R	99.47	96.20	99.77
e	99.92	99.90	99.92
j	99.47	96.99	99.70
A	98.87	93.14	99.36
a	99.87	99.07	99.94
J	99.68	98.23	99.81
S	99.81	99.81	99.81
V	99.49	96.59	99.75
E	99.98	99.90	99.98
F	99.5	97.77	99.66
Average	99.60	97.56	99.78

It is worth noting that the method in this work is not limited to the *Cascade RCNN* object detector, but applicable to other two-stage detectors or one-stage detectors. We could choose the appropriate detector as the basic model according to your needs.

## Discussion

Traditional ECG analysis methods require independent QRS complex detection that causes that the effectiveness of feature extraction and classification are highly dependent on the earlier detection accuracy. Error detection and missing detection can have a negative impact on feature extraction and eventually cause wrong classification results.

On the one hand, the morphology of ECG varies from person to person, even the same individual at different times, and the signals are easily disturbed by noises, which resulting in the difficulty of QRS complex detection. On the other hand, the QRS complex detection process is separate, so it is hard to achieve an end-to-end overall optimization through model training. It takes much time and effort, which is not beneficial for real-time testing.

This work avoids independent QRS complexes detection, which decouples the high dependence of feature extraction and heartbeats classification on detection accuracy.

### Overall Classification Performance

In order to take the position information as an object learned by the model, this work converts one-dimensional signals into two-dimensional images whose QRS complex position is annotated by automatic annotation algorithm. The positioning and classification tasks are unified in this work.

This work focuses on the interpretation of beat-level. Especially for an ECG signal containing multiple types of heartbeats such as bigeminy and trigeminy, we can get a detailed category of each beat for further analysis, rather than only the existence of abnormality by just classifying a signal over a long period of time.

In general, this work shows good performance in the beat-level classification of intra-patient paradigm. Table 4 shows a comparison with previous work. It can be seen that our method achieves the best results in both average classification accuracy and specificity. The sensitivity is slightly lower than some work which has fewer categories of interpretation. Obviously, our method is more advantageous in the number of categories.

**Table 4:** The performance of our proposed method compared with previous work.

**Work**	**Classes_n**	**Acc (%)**	**Sen (%)**	**Spe (%)**
Zhou et al. (36)	4	98.51	94.41	98.45
Hou et al. (37)	5	99.45	98.63	99.66
Wan et al. (38)	5	99.1	–	–
Ullah et al. (39)	8	99.11	97.91	99.61
Wang (40)	2	97.4	97.9	97.1
Chen et al. (41)	6	99.32	97.75	99.51
Niu et al. (42)	3	96.4	–	–
Houssein et al. (43)	4	98.26	97.43	–
Naz et al. (44)	4	97.6	–	–
This work	12	99.60	97.56	99.78

### The Importance of Object Detector

The human visual system is fast and accurate. At a glance, you can immediately know what the objects in the image are, where they are, and how they interact. We apply computer vision knowledge to ECG abnormality analysis, and use the object detection framework to directly predict the bounding boxes and class probabilities from the heartbeat images. During training and testing, the model can see the entire heartbeat image, so it implicitly encodes contextual information about the class and appearance to detect foreground objects more accurately, as well as directly optimizes the detection performance end-to-end.

This work constructs an object detection framework for heartbeat images based on *Cascade RCNN*, which consists of a series of cascading detection networks. Each detection network is trained on positive and negative samples based on different *IoU* thresholds. The output of the former network serves as the input to the latter, which is a stage-by-stage training method. The detector of each stage focuses on detecting the proposals whose *IoU* are in a certain range. The detection effect gets better and better since the output *IoU* is generally larger than the input *IoU*. We take a part of the heartbeat images as samples; Table 5 shows the detection accuracy of *Faster RCNN* (51) and *Cascade RCNN* on the four categories recommended by AAMI. Obviously, *Cascade RCNN* is more effective, whose *mAP@0.5* value is about 2 percent higher than *Faster RCNN*.

**Table 5:** Comparison of detection performance between Faster RCNN and Cascade RCNN.

		**Faster RCNN**			**Cascade RCNN**		
**Class**	**gts**	**dets**	**recall**	**AP**	**dets**	**recall**	**AP**
N	943	948	91.7	90.5	930	92.5	91.4
SVEB	934	931	92.9	91.6	937	95.0	94.1
VEB	882	950	97.7	97.0	895	96.9	96.3
F	238	217	83.6	83.1	235	88.7	87.8
mAP@0.5		90.5			92.4		

*gts, the number of ground truths; dets, the number of objects the model detects; recall, the ratio of true positive objects detected to all positive objects; AP, average precision of single category, calculated by PR curve; mAP@0.5, mean Average Precision (IoU = 0.5)*.

### The Effectiveness of Automatic Location Annotation

As we all known that the annotations play an important role in an object detection task, but only about 200–300 images can be processed per hour if we manually annotate images one by one like the similar work (51, 52). Obviously, the high human cost will discourage users and hardly expand to larger datasets, which deeply reduce the value of this kind of method.

A core strategy in this work is to automatically annotate the QRS complex position of the heartbeat images, which avoids the extremely time-consuming manual labeling process. Thousands of images can be annotated in just a few minutes, so instead of fixed lengths like one beat length (51) or 10 s (52), we can easily get the signal sequences of different lengths, as shown in Figure 4, which is beneficial for testing samples of unknown length.

**Figure 9:**
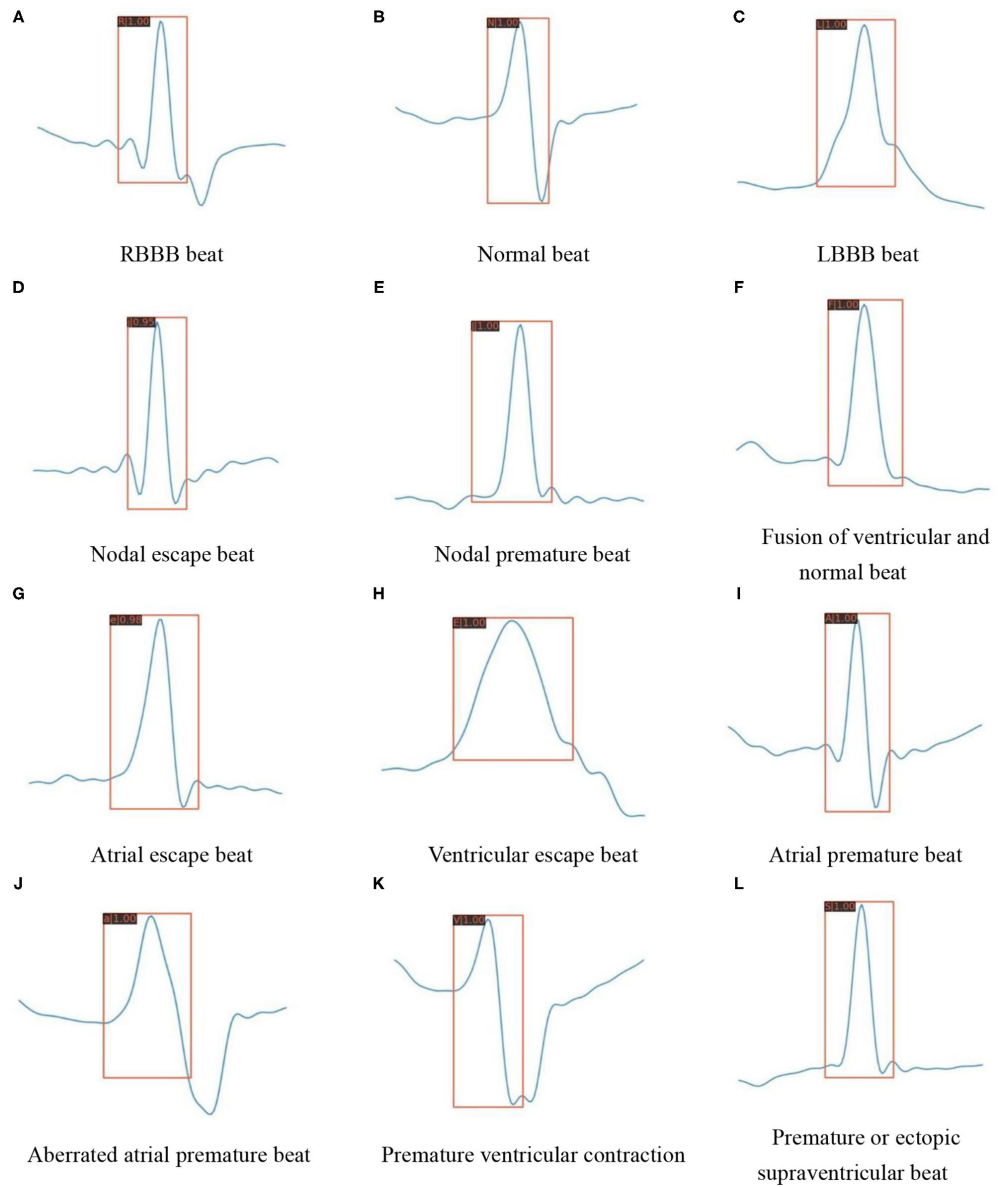
Detection results of the proposed model on various heartbeat images. **(A)** RBBB beat. **(B)** Normal beat. **(C)** LBBB beat. **(D)** Nodal escape beat. **(E)** Nodal premature beat. **(F)** Fusion of ventricular and normal beat. **(G)** Atrial escape beat. **(H)** Ventricular escape beat. **(I)** Atrial premature beat. **(J)** Aberrated atrial premature beat. **(K)** Premature ventricular contraction. **(L)** Premature or ectopic supraventricular beat.

Except for the improved classification accuracy as described in section Overall Classification Performance, the annotation is also essential for reducing the complexity during the inference stage. In the testing mode of traditional methods, as shown in Figure 1A, the preprocessed signals still need to perform the same process including QRS detection and feature extraction as in the training mode. The training stage does not facilitate the inference stage. The proposed method spends some labeling costs during the training stage, while in the testing mode, as shown in Figure 1B, the preprocessed signals need only be fed into the pretrained model as images. Figure 9 visualizes the prediction results for different categories of heartbeats; the model could output both the position of the QRS complex in the form of a rectangular box and the heartbeat category with a confidence score.

## Conclusion and Future Perspective

In this work, we propose a beat-level interpretation method based on object detection. We use a convolutional neural network as a feature extractor for two-dimensional heartbeat images, without complex manual design to extract features. Most importantly, this work abandons the previous mode of separate QRS complex detection and heartbeats classification, the ground truth of QRS complex is marked by automatic annotation algorithm, which is also regarded as the object, the model can learn like category information. The classification and regression branches of the object detector unify the localization and classification tasks, achieving an end-to-end optimization as well as decoupling the high dependence on the R-peak detection. We evaluate the performance on the MIT-BIH database, our method is superior to most advanced research even if the number of categories is as many as 12. The average accuracy is 99.60%, the average sensitivity is 97.56%, and the average specificity is 99.78%. In addition, since the independent and time-consuming QRS complex detection process is abandoned during the inference stage, our method is expected to be adopted in real-time monitoring systems to bring convenience to patients with cardiac abnormalities in the future. Of course, the MIT-BIH database contains too few patients to support the classification of inter-patient paradigm (53); the method in this work can be extended to the inter-patient paradigm when the beat-level annotations of more patients are obtained in the future.

## Data Availability Statement

Publicly available datasets were analyzed in this study. This data can be found here: physionet.org/content/mitdb/1.0.0/.

## Author Contributions

MK and X-FW contributed to conception and design of the study. MK wrote the codes and ran experiments on the relevant dataset and wrote the first draft of the manuscript. X-FW performed the statistical analysis. JX help to optimize the detection network. HT and T-LR supervised the whole work. X-FW, JX, HT, and T-LR refined the manuscript. All authors contributed to manuscript revision, read, and approved the submitted version.

## Funding

This work was supported by the National Natural Science Foundation of China (Grant 62022047, Grant 61874065, Grant U20A20168, and Grant 51861145202), Beijing Natural Science Foundation under Grant M22020, the National Key R&D Program under Grant 2016YFA0200400, in part by Fok Ying-Tong Education Foundation under Grant 171051, in part by Beijing National Research Center for Information Science and Technology Youth Innovation Fund (BNR2021RC01007), in part by State Key Laboratory of New Ceramic and Fine Processing Tsinghua University (No. KF202109), in part by the Research Fund from Beijing Innovation Center for Future Chip and the Independent Research Program of Tsinghua University under Grant 20193080047, and Tsinghua-Foshan Innovation Special Fund (2021THFS0217).

## Conflict of Interest

JX was employed by Ping An Technology (Shenzhen) Co. Ltd. The remaining authors declare that the research was conducted in the absence of any commercial or financial relationships that could be construed as a potential conflict of interest.

## Publisher's Note

All claims expressed in this article are solely those of the authors and do not necessarily represent those of their affiliated organizations, or those of the publisher, the editors and the reviewers. Any product that may be evaluated in this article, or claim that may be made by its manufacturer, is not guaranteed or endorsed by the publisher.
